# Generation of vascularized human cardiac organoids for 3D *in vitro* modeling

**DOI:** 10.1016/j.xpro.2023.102371

**Published:** 2023-06-28

**Authors:** Holly K. Voges, Richard J. Mills, Enzo R. Porrello, James E. Hudson

**Affiliations:** 1 QIMR Berghofer Medical Research Institute, Brisbane, QLD 4006 Australia; 2 Murdoch Children’s Research Institute, The Royal Children’s Hospital, Melbourne, VIC 3052 Australia; 3 Department of Paediatrics, School of Medicine, Dentistry and Health Sciences, The University of Melbourne, Melbourne, VIC 3052, Australia; 4 School of Biomedical Sciences, The University of Queensland, Brisbane, QLD 4072, Australia; 5 Novo Nordisk Foundation Center for Stem Cell Medicine, Murdoch Children’s Research Institute, Melbourne, VIC 3052, Australia; 6 School of Biomedical Sciences, Queensland University of Technology, Kelvin Grove, QLD 4000, Australia; 7 Department of Anatomy and Physiology, School of Biomedical Sciences, The University of Melbourne, Melbourne, VIC 3052, Australia; 8 Melbourne Centre for Cardiovascular Genomics and Regenerative Medicine, The Royal Children’s Hospital, Melbourne, VIC 3052, Australia

**Keywords:** Cell culture, Cell Differentiation, Tissue Engineering

## Abstract

Here, we provide a protocol for next-generation human cardiac organoid modeling containing markers of vascularized tissues. We describe steps for cardiac differentiation, harvesting cardiac cells, and generating vascularized human cardiac organoids. We then detail downstream analysis of functional parameters and fluorescence labeling of human cardiac organoids. This protocol is useful for high throughput disease modeling, drug discovery, and providing mechanistic insight into cell-cell and cell-matrix interactions.

For complete details on the use and execution of this protocol, please refer to Voges et al.^1^ and Mills et al.^2^

## Highlights

• A protocol for differentiating cardiac cells from human pluripotent stem cells
• Generation of vascularized human cardiac organoids
• Analysis of organoid contractile force and immunofluorescence

## Abstract (editor highlights)

Publisher’s note: Undertaking any experimental protocol requires adherence to local institutional guidelines for laboratory safety and ethics.

## Graphical abstract

**Figure:**
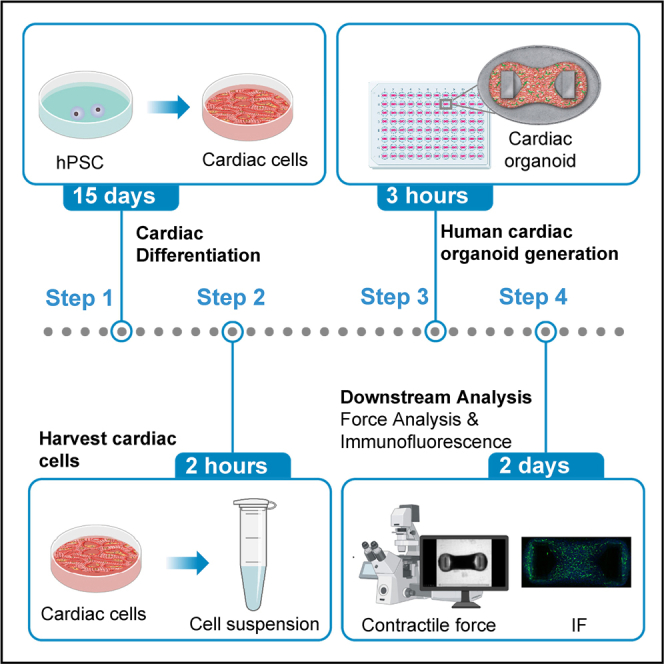

## Before you begin

Before using the methods described in this protocol, it is essential that the users obtain relevant ethical approval for the culture of human pluripotent stem cell lines.

Also note that this protocol is optimized for feeder-free pluripotent stem cell culture, so adaptation of pluripotent stem cell lines may be required.

SU-8 wafer manufactured in a fabrication facility is required before you begin. Computer-aided design (CAD) file attached.

### Preparation of Matrigel™ coated plates

**Timing: 7 h**

***Note:*** The following steps should be conducted in a biosafety cabinet.

1. Thaw a bottle of LDEV-Free Matrigel™ (10 mL) on ice for 6 h.
2. On ice, aliquot 500 μL of Matrigel™ into sterile Eppendorf tubes.
3. Store aliquots at −20°C until required.
4. Prepare a working solution of Matrigel™:
  a. Thaw aliquot on ice for 1 h until liquid.
  b. Aliquot 49.5 mL PBS (minus magnesium and calcium) and store on ice until ready to mix.
  c. Check Matrigel™ dilution factor as per manufacturer’s instructions. E.g., Add 500 μL (or as instructed) of Matrigel™ to ice-cold PBS for a 1:50 dilution (v/v).
  d. Rinse Eppendorf with PBS to transfer entire aliquot.

***Note:*** working solution of Matrigel™ can be stored at 4°C for 2 weeks.

5. Coat each T25 with 2.5 mL of Matrigel™ as required.
6. Leave coated flasks in biosafety cabinet for a minimum of 1 h before use.

***Note:*** Alternatively, transfer coated flask to cell culture incubator at 37°C for minimum 30 min before use, or pre-coat the flask the night before and store in the fridge at 4°C.

**CRITICAL:** It is critical to use pre-chilled PBS and ensure Matrigel™ is always kept at 4°C otherwise it will quickly gel.

**CRITICAL:** When scaling to other culture vessel sizes it is critical that the surface area to volume ratio is maintained.

**CRITICAL:** For some batches of Matrigel™ with very different concentrations to standard, the dilution may require adjustment. See product data sheet for further details.

### Maintenance of human pluripotent stem cells

**Timing: 7 days**

7. Culture human pluripotent stem cell lines on Matrigel™ coated T25 flasks (see steps 1–6). This protocol can be used on both human embryonic stem cell and human induced pluripotent stem cell lines.
8. On Day -4 (Friday) prepare T25 flask for differentiation:
  a. Aspirate mTeSR™ PLUS medium from T25 culture flask. Flask should be ∼70% confluent.
  b. Wash cells with 3–5 mL of warmed (37°C) PBS (without Calcium or Magnesium).
  c. Aspirate PBS.
  d. Gently add 2 mL of room temperature (15°C–25°C) ReLeSR™ to cover the cells.
  e. Aspirate the ReLeSR™ within 1 min (∼50s) so that the culture is left with a thin film of ReLeSR™.
  f. Incubate at 37°C for 5–8 min depending on the cell line.
  g. Tilt the flask and use 3 mL mTeSR™ PLUS to wash the colonies to the bottom corner of the flask.
    ***Note:*** No rock inhibitor is used for passaging hPSCs.
  h. If colonies do not detach easily hold the plate firmly and tap the side of the flask firmly for 30–60 s.
  i. Slowly transfer the cell suspension to a sterile 15 mL canonical tube using a 10 mL serological pipette to maintain aggregates.
  j. Prepare aliquot for counting.
  k. Work carefully and do not over pipette the cell suspension to maintain aggregates.
9. Work quickly to count cells within aggregates using a hemocytometer.
  a. Add 10 mL of pre-warmed mTeSR™ PLUS (37°C) to a new Matrigel™ coated T25 flask.
  b. Transfer required amount from the cell suspension canonical tube into the new flask, plating 400,000 cells per T25 flask.
  c. Gently move the flask from side to side to ensure cells spread evenly in the flask.
  d. Return cells to 37°C 5% CO2 for the weekend (∼72 h).
  e. On Monday change the medium with 5mL of fresh, pre-warmed media:
    i Culture maintenance flasks of human pluripotent stem cells in mTeSR™ PLUS.
    ii Culture flasks going into differentiation in mTeSR™-1, as the stabilized growth factors in mTeSR™ PLUS can interfere with the first few days of differentiation.
  f. On Tuesday passage the maintenance flasks of the human pluripotent stem cells as per a-o, with daily 5 mL medium changes with mTesr1 until Friday.
    ***Note:*** Each cell line will have variations in initial seeding density depending on growth rate (typically 300,000–500,000 per T25). Seed cells so that they are ∼70% confluent by Day 0 of differentiation. Rock inhibitor is not used at any point to passage stem cell lines.

### Manufacture of silicone inserts for 96 well plates

**Timing: 2 days**

10. SU-9 wafers are fabricated using photolithography. For details please see relevant publication.^3^
11. Pre-heat oven to 65°C and clean SU-8 wafer with 80% ethanol, followed by water and dry with compressed air. Place clean SU-8 wafer in clean 15 mm petri dish (Figure 1A).

**Figure 1:**
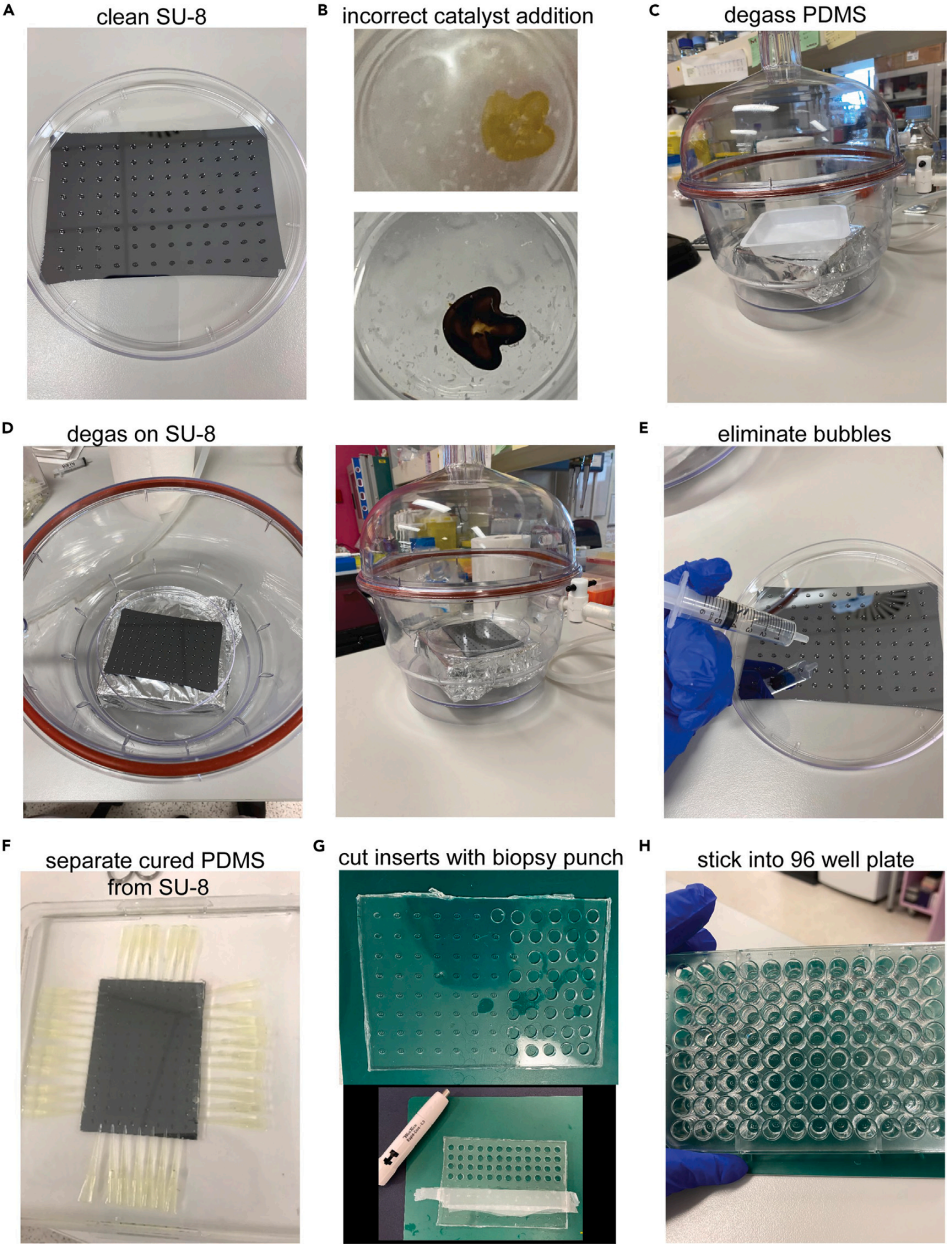
Preparation of PDMS culture inserts (A) Preparation of custom SU-8 wafer for PDMS casting. Wafer thickness 1 mm, 0.7 mm pillar height, 0.2 mm pillar width and 0.5 mm pillar length. Cleaned and transferred to 15mm petri dish. (B) Mixing of crosslinker and PDMS base agent with example of incorrect catalyst addition leaving yellow crosslinked debris. (C) Degassing of PDMS in a weigh boat. (D) Degassing of PDMS coating the SU-8 wafer. (E) Final inspection of PDMS and removal of stubborn bubbles with air extrusion. (F) After PDMS is cured in oven, separation of PDMS from SU-8 is required. SU-8 is transferred to a large square petri dish and covered in 80% Ethanol. Pipette tips are used to slowly separate the cured PDMS layer. (G) Once the PDMS sheet is removed from SU-8, cleaned and sealed with sticky tape, a 0.5 mm biopsy punch is used to cut individual inserts. (H) PDMS inserts are transferred and glued into a 96 well culture plate using degassed PDMS glue and left to cure overnight (∼18 h).
12. Mix PDMS elastomer (Sylgard 184) with the curing agent at a 10 to 1 ratio (w/w):
  a. E.g., Weigh out 45 g of PDMS base agent into large weigh boat slowly due to viscous nature.
  b. Weigh 4.5 g of PDMS curing agent.
  c. Mix thoroughly with spatula for several min.
13. Once mixed thoroughly, add platinum catalyst at 0.555 μL per gram of total combined PDMS elastomer and mix thoroughly with a spatula for several min.

**CRITICAL:** To ensure even distribution of the catalyst, add slowly while moving the pipette tip through the PDMS elastomer. Uneven catalyst incorporation will result in premature polymer crosslinking, visualized as yellow polymer (Figure 1B), if this occurs discard mixture and repeat steps 10 & 11.

**CRITICAL:** The catalyst is a hazardous chemical and the proper safety precautions should be taken.

14. To remove bubbles from the PDMS elastomer, place weigh boat in the vacuum desiccator on a flat surface for 30 min with regular re-pressurizing (Figure 1C).

***Alternatives:*** elastomer can be combined in any vessel, but a large weigh boat maximizes the surface area and increases the speed of removing bubbles.

15. Once bubbles are removed, add 42 g (of 45 g made up in step 11) of PDMS elastomer to the SU-8 wafer on the precision balance and return to the vacuum desiccator for 40 min (Figure 1D).
16. Visually inspect that no bubbles have remained in the pillar forming holes of the SU-8 wafer. Use syringe to expel air directly above stubborn bubbles to remove (Figure 1E).
17. Place SU-8 wafer + PDMS in oven for 35 min to cure. Once cured, remove the SU-8 wafer and store at room temperature (15°C–25°C) overnight (∼18 h).

**CRITICAL:** For successful plate fabrication, ensure plate is level in the oven by using a spirit level. Temperature of curing and ratio of base/curator agent will impact elastic modulus of the resulting polymer and therefore the downstream force analysis.

18. On day 2, cut and remove excess PDMS from around the SU-8 wafer + PDMS edges with a scalpel.
19. Transfer SU-8 wafer into 245 mm square tissue culture dish and cover with 80% ethanol.

***Alternatives:*** any large flat dish can be used that has enough room to place pipette tips around the perimeter of the SU-8 wafer.

20. Slide pipette tips between SU-8 wafer and cured PDMS elastomer to slowly separate.
  a. Once PDMS is removed from the wafer, place on a cutting mat as in step 21.
  b. Meanwhile dry the SU-8 wafer with a non-particle producing tissue wipe and compressed air before storing (Figure 1F).
21. Prepare 2 g of elastomer mixture at a ratio of 10 to 1 base to curing agent in a 50 mL canonical and mix thoroughly.
  a. Centrifuge at 300 × *g* for 3 min to quickly remove bubbles.
  b. Using a P200 pipette, transfer enough PDMS to cover the surface of the well (approx. 10 μL) in each of a new 96 well culture plate. This will be the PDMS glue.
22. Clean the back and pillar side of the PDMS sheet with sticky tape to remove dust and debris, and place on cutting mat with the pole side facing up (Figure 1G).
23. Using a 6 mm biopsy punch, cut individual well inserts out of the sheet and use the biopsy punch to also release and stick down one per well of 96 well plate with the PDMS glue as in step 20 (Figure 1G).
24. Store plate at room temperature (15°C–25°C) overnight (∼18 h) to ensure well inserts cure to the 96 well plate before use (Figure 1H).
  a. Day before use, cover plate in 80% ethanol inside biosafety hood for > 2 h and sterilize with 2 × 20 min UV exposures.
  b. Rotate plates 90 between the UV exposures to ensure complete sterilization. .

### Stock cell culture reagents: Growth factors, small molecules, and supplements

**Timing: 6 h**

25. Prepare stock reagents in a biosafety cabinet using aseptic technique. Ahead of time, ensure that 1.5 mL Eppendorf tubes and 0.2mL PCR tubes have been sterilized.
26. L-ascorbic acid 2 phosphate (AA2P):
  a. Quick spin the vial to ensure no product is lost.
  b. Reconstitute vial to 200 mM in milli-Q water.
    i. E.g., Add 43.15 mL milli-Q water to 2.5 g stock and mix well.
  c. Filter sterilize into 50 mL conical tube.
  d. Prepare 500 μL aliquots in sterilized 1.5 mL Eppendorf tubes.
  e. Store at −20°C.

***Note:*** Aliquots are stable when stored at −20°C for 12 months. Freeze thaw is not recommended for product stability.

27. Human Bone Morphogenetic Protein 4 (BMP4):
  a. Quick spin the vial to ensure no product is lost.
  b. Reconstitute vial to 500 μg/mL in 4 mM hydrochloric acid.
    i. E.g., add 100 μL 4 mM Hydrochloric acid to 50 μg BMP4.
  c. Dilute this solution in 0.1% (w/v) bovine serum albumin (BSA) in milli-Q water to 20 μg/mL.
  d. Prepare 50 μL aliquots in sterilized 0.2 mL PCR tubes.
  e. Transfer PCR tubes to 50 mL conical tube and store at −20°C.

***Note:*** Aliquots are stable when stored at −20°C for 3 months. Freeze thaw is not recommended for product stability. We keep thawed reagents at 4°C for 1 week maximum.

28. Human Activin-A:
  a. Quick spin the vial to ensure no product is lost.
  b. Reconstitute vial to 36 μg/mL in 0.1% (w/v) BSA in PBS.
    i. E.g., Add 1.38 mL of 0.1% BSA in PBS to 50 μg stock and mix well.
  c. Prepare 50 μL aliquots in sterilized 0.2 mL PCR tubes.
  d. Transfer PCR tubes to 50 mL conical tube and store −20°C.

***Note:*** Aliquots are stable when stored at −20°C for 3 months. Freeze thaw is not recommended for product stability. Thawed reagents are stable at 4°C for 1 month.

29. Human basic fibroblast growth factor (FGF-2):
  a. Quick spin the vial to ensure no product is lost.
  b. Reconstitute vial to 20 μg/mL in 0.1% (w/v) BSA in PBS.
  c. E.g., Add 1.25 mL 0.1% BSA in PBS to 25 μg stock and mix well.
  d. Prepare 50 μL aliquots in sterilized 0.2 mL PCR tubes.
  e. Transfer PCR tubes to 50 mL conical tube and store −20°C.

***Note:*** Aliquots are stable when stored at −20°C for 3 months. Freeze thaw is not recommended for product stability. We keep thawed reagents at 4°C for 1 week maximum.

30. CHIR99021.
  a. Quick spin the vial to ensure no product is lost.
  b. Reconstitute vial to 10 mM in dimethyl sulfoxide (DMSO).
    i. E.g., Add 2.15 mL DMSO to 10 mg stock and mix well.
  c. Prepare 20 μL aliquots in sterilized 0.2 mL PCR tubes.
  d. Transfer PCR tubes to 50 mL conical tube and store −20°C.

***Note:*** Aliquots are stable when stored at −20°C for 3 months. Freeze thaw is not recommended for product stability. We keep thawed reagents at 4°C for 1 week maximum.

31. IWP-4:
  a. Quick spin the vial to ensure no product is lost.
  b. Reconstitute vial to 5 mM in DMSO.
    i. E.g., Add 4.03 mL DMSO to 10 mg stock and mix well.
  c. May require a freeze-thaw and mixing at 37°C to properly get into solution.
  d. Prepare 50 μL aliquots in sterilized 0.2 mL PCR tubes.
  e. Transfer PCR tubes to 50 mL conical tube and store −20°C.

***Note:*** Aliquots are stable when stored at −20°C for 3 months. Another freeze thaw is not recommended for product stability. We keep thawed reagents at 4°C for 1 week maximum.

32. Human platelet-derived growth factor BB (PDGF-BB):
  a. Reconstitute vial to 20 μg/mL in 0.1% BSA in PBS.
    i. E.g., Add 2.5 mL 0.1% BSA in PBS to 50 μg PDGF-BB.
  b. Filter sterilize solution into 50 mL tube.
  c. Prepare 20μL aliquots in sterilized 0.2 mL PCR tubes.
  d. Transfer PCR tubes to 50 mL conical tube and store −20°C.

***Note:*** Aliquots are stable when stored at −20°C for 3 months. Freeze thaw is not recommended for product stability. We keep thawed reagents at 4°C for 1 week maximum.

33. Insulin, human recombinant:
  a. Stock vial comes at 4 mg/mL in zinc solution.
  b. Prepare 2–5 μL aliquots in sterilized 0.2 mL PCR tubes.
  c. Transfer PCR tubes to 50 mL conical tube and store −20°C.

***Note:*** Aliquots are stable when stored at −20°C for 12 months. Freeze thaw is not recommended for product stability. For use, dilute insulin 1:500 in DMEM and use this fresh in weaning medium.

34. B-27 supplement (both with and without insulin):
  a. Thaw stock 10 mL supplement bottles at room temperature (15°C–25°C).
  b. Aliquot supplements into 1 mL aliquots in 1.5 mL Eppendorf tubes.
  c. Store at −20°C. Freeze thaw is not recommended for product stability. Thaw aliquot immediately before use.
35. Palmitic acid:
  a. Reconstitute vial to 0.1 M in DMSO.
    i. E.g., Add 19.4 mL DMSO to 500 mg Palmitic acid.
  b. Prepare 100 μL aliquots in sterilized 0.2 mL PCR tubes.
  c. Transfer PCR tubes to 50 mL conical tube and store −20°C.

***Note:*** Aliquots are stable when stored at −20°C for 12 months. Freeze thaw is not recommended for product stability. We keep thawed reagents at 4°C for 1 week maximum.

36. D-glucose at 1 M:
  a. Reconstitute D-glucose to 1 M in milli-Q water.
    i. E.g., Add 50 mL milli-Q water to 9 *g* D-glucose.
  b. Filter sterilize solution into 50 mL conical tube.
  c. Store at 4°C for up to 3 months.
37. Aprotinin:
  a. Reconstitute vial to 33 mg/mL in PBS.
    i. E.g., Add 15.1 mL PBS to 500 mg stock.
  b. Aliquot stock solution in 50 μL into sterilized 1.5 mL Eppendorf tubes.
  c. Transfer PCR tubes to 50 mL conical tube and store −20°C.

***Note:*** A aliquots are stable when stored at −80°C for 6 months. Freeze thaw is not recommended for product stability. Use on day of thaw.

38. Collagenase I:
  a. Make up 20% fetal bovine serum (FBS) in PBS with calcium and magnesium (+/+).
    i. E.g., Add 10 mL FBS to 40 mL PBS (+/+).
  b. Reconstitute collagenase I to 0.2% (w/v).
    i. E.g., Add 50 mL 20% FBS in PBS to 100 mg of collagenase I.
  c. Filter sterilize solution into 50 mL conical tube.
  d. Aliquot stock solution in 5 mL into 15 mL conical tubes.
  e. Store at −20°C.

***Note:*** Aliquots are stable when stored at −20°C for 12 months. Freeze thaw is not recommended for product stability. Use on the day of thaw.

***Note:*** Occasionally the concentration of the collagenase I needs to be adjusted based on the enzymatic activity of a particular batch.

39. 3% bovine serum albumin (BSA):
  a. Make up 3% BSA in PBS.
    i. E.g., weigh 3 *g* in 100 mL PBS.
  b. Filter sterilize solution into 50 mL tube.
  c. Store at 4°C for up to 1 month.
40. DMEM for organoids:
  a. Make up 10X DMEM in milli-Q water.
    i. E.g., Weigh 1.996 *g* of DMEM powder.
    ii. Add 740 mg NaHCO3.
    iii. Re-suspend in 20mL milli-Q water.
    iv. Heat solution at 37°C to help dissolve the solution.
  b. Adjust pH to 7.2.
  c. Filter sterilize solution into 50 mL tube.
  d. Store at 4°C for up to 1 month.
41. NaOH for organoids:
  a. Make up 0.1 M NaOH in milli-Q water.
    i. E.g., Add 187.5 mg to 43.7 mL milli-Q water.
    ii. Re-suspend solution.
  b. Filter sterilize solution into a sterile 50 mL conical tube.
  c. Store at 4°C for up to 1 month.
42. Conjugation of Palmitate to B27 supplement, minus insulin:
  a. Heat 2 × 1 mL aliquots of B27- and 1 × 100 μL aliquot of palmitate in water bath at 37°C.
  b. Once warm, add 25 μL of warmed palmitate to each 1 mL B27- aliquot.
    ***Note:*** Aliquots should be completely thawed and warmed before addition or the palmitate will not go into solution.
  c. Shake at 37°C for 2 h at 1250x RPM covered with aluminum foil.
    ***Note:*** Solution should be clear with no precipitate to indicate proper conjugation.
  d. Add conjugated B27- to maturation medium (described below) immediately, or store at 4°C for up to 1 week.

## Key resources table

**Table:** 

REAGENT or RESOURCE	SOURCE	IDENTIFIER
**Antibodies**		

Mouse IgG_1_ anti-human CD31, use 1:100	Dako	RRID: AB_2114471
Mouse IgG_2a_ anti-CD90/Thy1 Mab, use 1:500	R&D Systems	RRID: AB_2203306
Rabbit anti-cardiac Troponin T, use 1:400 for immunostaining	Abcam	RRID: AB_956386
Mouse IgG_1_ anti-α-actinin, use 1:1000	Sigma	RRID: AB_476766
Mouse anti-PDGF Rβ Monoclonal Antibody, use 1:250	R&D Systems	Cat# MAB1263
Mouse IgG_2B_ anti-VE-cadherin antibody, use 1:100	R&D Systems	RRID: AB_2260374
Goat anti-Mouse IgG2a Cross-Absorbed Secondary Antibody, Alexa Fluor 555, use 1:400	Thermo Fisher Scientific	RRID: AB_2535776
Goat anti-Rabbit IgG (H + L) Cross-Absorbed Secondary Antibody, Alexa Fluor 633 use 1:400	Thermo Fisher Scientific	RRID: AB_2535731
Goat anti-Mouse IgG (H + L) Cross-Absorbed Secondary Antibody, Alexa Fluor 647, use 1:400	Thermo Fisher Scientific	RRID: AB_2535804
Alexa Fluor 488 Goat Anti-Mouse IgM, use 1:400	Life Technologies	A21042
Hoechst33342, trihydrochloride, trihydrate, use 1:1000	Life Technologies	H3570

**Chemicals, peptides, and recombinant proteins**		

Recombinant Human BMP-4	R&D Systems	314-BP-050/CF
IWP-4	Stem Cell Technologies	72554
Recombinant Human FGF basic (146a)	R&D Systems	233FB025CF
CHIR99021	Stemgent	72054
Human platelet-derived growth factor-BB (PDGF-BB)	R&D Systems	220BB050
Recombinant Human Activin-A	R&D Systems	338-AC-050
L-Ascorbic acid 2-phosphate sesquimagnesium salt hydrate (AA2P)	Sigma	A8960-5G
Penicillin-streptomycin (10,000U/mL)	Thermo Fisher Scientific	15140-122
ReLeSR™	Life Technologies	05872
RPMI 1640 Medium, GlutaMAX	Life Technologies	61870127
DMEM, no glucose, no glutamine, no phenol red	Thermo Fisher Scientific	A1443001
MEMα, GlutaMAX Supplement, no nucleoside	Life Technologies	32561-037
B27 Serum-Free Supplement (50X) liquid	Life Technologies	17504-044
B27 supplement, minus insulin	Life Technologies	A1895601
mTESR-1™ complete medium kit	Stem Cell Technologies	85857
mTESR™-PLUS complete medium kit	Stem Cell Technologies	100-0276
Fetal Bovine Serum, qualified, Australian Origin	Life Technologies	10099141
Matrigel™ Basement Membrane Matrix, LDEV-Free	Corning	354234
Collagenase type 1	Sigma-Aldrich	C0130
Trypsin-EDTA (0.25%), phenol red	Life Technologies	25200072
Phosphate buffered saline (minus magnesium and calcium)	Life Technologies	10010023
Insulin, human recombinant 4 mg/mL	Thermo Fisher Scientific	12585014
Palmitic acid	Sigma	P0500
D-glucose	Sigma	G8270
Aprotinin	Med Chem Express	P0017
Dimethyl sulfoxide (DMSO)	Merck	67-68-5
Sylgard 184 Silicone elastomer kit, polydimethylsiloxane (PDMS)	Dow Corning	761036
Platinum Catalyst (platinum-divinyltetramethyldisiloxane complex in xylene, low color (2.1–2.4% platinum))	Abcr GmbH	AB153234
SU-8 master	Nanofabrication Facility	
Cutting mat 140 × 89mm	ProSciTech	T984-M
Biopsy Puncher with plunger, 6mm	ProSciTech	T982-60
Surgical Scalpel Blade No 23	Swann-Morton	0210
Acid solubilized bovine collagen 1	Devro	01APA006
Sodium hydroxide	Chem-Supply	SA178
DMEM powder, low glucose, pyruvate	Thermo Fisher Scientific	31600-083
Matrigel™ Basement Membrane Matrix, LDEV-Free	Corning	354234
Bovine serum albumin	Sigma	A9418
ProLong™ Glass Antifade Mountant	Thermo Fisher Scientific	P36984
Triton™ X-100	Sigma	9036-19-5

**Experimental models: Organisms/strains**		

Human: embryonic stem cell line HES-3, female	WiCell	RRID: CVCL_7158
Human: embryonic stem cell line H9, female	WiCell	RRID: CVCL_JL69
Human: induced pluripotent stem cell line AA, male	CIRM hPSC Repository	CW30382A
Human: induced pluripotent stem cell line CC, female	CIRM hPSC Repository	CW30318C
Human: induced pluripotent stem cell line HH, female	CIRM hPSC Repository	CW30171HH1

**Software and algorithms**		

Pole tracking analysis	Mills et al.^3^	MATLAB m-files attached

**Other**		

Vacuum desiccator and chamber	Thermo Fisher	5311-0250PK
Drying oven		
Luer syringe, 50 mL, single use	Sarstedt Australia	94.6077.137

## Materials and equipment

• mTeSR™ stem cell medium: add 0.5% Penicillin-Streptomycin to supplement and base media.

[Store at 4°C until manufacturer’s expiration date].

• Immunohistochemistry blocking buffer: add 125 μL Triton-X and 2.5 mL FBS to PBS.

[Store at 4°C and use within 1 month].

**Table:** RPMI B27 (minus insulin) medium

Reagent	Final concentration	Amount
RPMI 1640 GlutaMAX	N/A	500 mL
Penicillin-Streptomycin	1%	5 mL
B27 supplement minus insulin	2%	
L-Ascorbic Acid 2-phosphate sesquimagnesium salt hydrate	200 μM	500 μL
**Total**	**N/A**	**515.5 mL**

Store at 4°C for 1 week maximum once the B27 is added. If the medium requirements are low, make base RPMI media without B27 and add the B27 in 50 mL batches as needed.

**Table:** RPMI B27 medium

Reagent	Final concentration	Amount
RPMI 1640 GlutaMAX	N/A	500 mL
B27 supplement	2%	10 mL
Penicillin-Streptomycin	1%	5 mL
L-Ascorbic Acid 2-phosphate sesquimagnesium salt hydrate	200 μM	500 μL
**Total**	**N/A**	**515.5 mL**

Store at 4°C for 1 week maximum once the B27 is added. If the medium requirements are low, make base RPMI media without B27 and add the B27 in 50 mL batches as needed.

**Table:** Cardiac Differentiation medium Day 0 -2 (volumes per T25)

Reagent	Final concentration	Amount
RPMI B27 minus insulin medium (above)	X	5 mL
Activin A	9 ng/mL	1.25 μL
BMP-4	5 ng/mL	1.25 μL
CHIR99021	1 μM	0.5 μL
FGF-2	5 ng/mL	1.25 μL
**Total**	**N/A**	**5 mL**

Make fresh with every use.

**Table:** Organoid Matrix Mixture (110 organoids + 10%)

Reagent	Final concentration	Amount
Collagen I @6.4 mg/mL	0.091 mg per tissue	172 μL
10x DMEM	0.121 μL per μL collagen I	20.6 μL
0.1 M NaOH	0.15 μL per μL collagen I	25.8 μL
Matrigel™	9%	38.1 μL
Cells	6.6 × 10^6^	167 μL
**Total**	**N/A**	**423.5 μL**

Prepare on ice **immediately** before adding the cell suspension. May require adjustment if stock collagen I is supplied at a different concentration. Mix collagen I prior to use or aliquoting as it may slightly settle into a more viscous and less viscous phase over long time frames.

**Table:** Organoid harvest medium

Reagent	Final concentration	Amount
MEMα GlutaMAX	N/A	45 mL
Penicillin-Streptomycin	1%	0.5 mL
L-Ascorbic Acid 2-phosphate sesquimagnesium salt hydrate	200 μM	50 μL
Fetal Bovine Serum	10%	5 mL
**Total**	**N/A**	**50 mL**

Store at 4°C for 2 weeks.

**Table:** Serum Free Medium

Reagent	Final concentration	Amount
MEMα GlutaMAX	X	47.9 mL
L-Ascorbic Acid 2-phosphate sesquimagnesium salt hydrate	200 μM	50 μL
Penicillin-Streptomycin	1%	500 μL
B27 supplement	4%	2 mL
FGF-2	10 ng/mL	25 μL
PDGF-BB	10 ng/mL	25 μL
**Total**		**50 mL**

Store at 4°C and use within 3 months.

**Table:** Maturation Medium

Reagent	Final concentration	Amount
DMEM (minus glucose, phenol red, glutamine and sodium pyruvate)	X	46.85 mL
B27 supplement, minus insulin∗	2X	2 mL
Penicillin-Streptomycin	1%	500 μL
Glutamax	1X	500 μL
Ascorbic Acid	200 μM	50 μL
Palmitic Acid∗	100 μM	50 μL
D-Glucose	1 mM	50 μL
Aprotinin	33 μg/mL	50 μL
FGF-2	10 ng/mL	25 μL
PDGF-BB	10 ng/mL	25 μL
**Total**	**N/A**	**50 mL**

Store at 4°C and use within 1 week once B27 and palmitate are added. ∗**Note** pre-conjugation step of palmitic acid to B27 supplement outlined in step 41.

**Table:** Weaning Medium

Reagent	Final concentration	Amount
DMEM (minus glucose, phenol red, glutamine and sodium pyruvate)	X	48 mL
B27 supplement, minus insulin∗	4X	4 mL
Penicillin-Streptomycin	1%	500 μL
Glutamax	1X	500 μL
Ascorbic Acid	200 μM	50 μL
D-Glucose	5.5 mM	276 μL
Insulin	1 nM	36.2 μL
Palmitic Acid∗	10 μM	5 μL
Aprotinin	33 μg/mL	50 μL
**Total**	**N/A**	**50 mL**

Store at 4°C and use within 1 week once B27 and palmitate are added. ∗**Note** pre-conjugation step of palmitic acid to B27 supplement outlined in step 41.

## Step-by-step method details

### Differentiation of cardiomyocytes and stromal cells from hPSCs

**Timing: 15 days**

This section achieves the differentiation of cardiac cell types over a 15-day protocol.

1. On Day 0 of differentiation, pluripotent stem cells should be ∼70% confluent. Prepare RPMI B27 minus insulin medium (table above):
  a. Prepare 5 mL RPMI/B27 minus insulin medium per T25 flask.
  b. Add final concentration 5 ng/mL BMP-4, 9 ng/mL Activin A, 5 ng/mL bFGF and 1 μM CHIR99021.
  c. E.g., add 1.25 μL BMP-4, 1.25 μL Activin A, 1.25 μL bFGF, and 0.5 μL CHIR99021 as per Cardiac differentiation medium Day 0–2 table.
    **CRITICAL:** Thaw growth factors before use. Add growth factors and small molecules immediately prior to use and add to warm medium (37°C).
  d. Wash with RPMI base media on Day 0 before adding differentiation media.
2. On Day 3, wash cell with RPMI base medium to remove cell debris:
  a. Prepare 10 mL RPMI/B27 minus insulin medium per T25.
  b. Add final concentration 5 μM IWP4.
  c. E.g., add 10 μL IWP4.

**CRITICAL:** Add IWP4 immediately prior to use and warm to 37°C.

3. On Day 6, prepare 49 mL supplemented RPMI base medium and add 1 mL B27 supplement, plus insulin:
  a. On Day 6, 8 and 10 prepare 5 mL RPMI/B27 (plus insulin) medium per T25 flask.
  b. Add 5 μM IWP4.

***Note:*** Cardiomyocyte beating is generally observed from day 10 onwards.

4. On Day 13, perform media change with RPMI/B27 (plus insulin) with no factors.
5. On Day 15, harvest cells for organoid generation as outline below.

### Harvest of differentiated cardiac cells for organoids

**Timing: 2 h**

This section describes the generation of human cardiac organoids.

6. Prepare PDMS culture plates from step 23 for use:
  a. Inside biosafety hood, ensure no residual ethanol remains in the well inserts.
  b. Coat inserts with 3% BSA in PBS (approx. 5–10 μL per well) for > 60 min at room temperature (15°C–25°C).

### Begin cell dissociation

7. Aspirate medium from T25.
8. Add 2.5 mL collagenase I (**step 36**) per T25 and incubate for 1 h at 37°C 5% CO2.
  a. Gently tap the side of flask every 10 min to help dissociation.
9. Add 7.5 mL PBS (no calcium, no magnesium) to T25 and titrate liquid and cells up and down ∼5 times to disrupt cells with a 10 mL serological pipette.
10. Collect cell solution into 50 mL conical tube.
11. Centrifuge at 300 × *g* for 3 min.
12. Aspirate supernatant.
13. Re-suspend cell pellet in 5 mL 0.25% Trypsin with EDTA per T25.
14. Gently agitate cells in water bath at 37°C for 10 min.
15. Neutralize enzyme with 5 mL organoid harvest medium (see Table above) per T25.
16. Filter cells through 100 μm Corning cell strainer and perform cell count.

### Human cardiac organoid fabrication

**Timing: 3 h**

17. Prepare PDMS culture plates for use in culture:
  a. Inside biosafety hood, aspirate 3% BSA (from step 6b) and ensure no residual liquid remains in the well inserts.

**CRITICAL:** Ensure insert has no remaining liquid or the organoid mixture will be subsequently diluted and compromised.

18. Perform cell counts and prepare cell aliquots per plate:
  a. For 1 × 96 well plate, calculate 110 cardiac organoids and 6.6 × 106 cells.
  b. Centrifuge cell suspension for 300 × *g* for 3 min.

**Figure 2:**
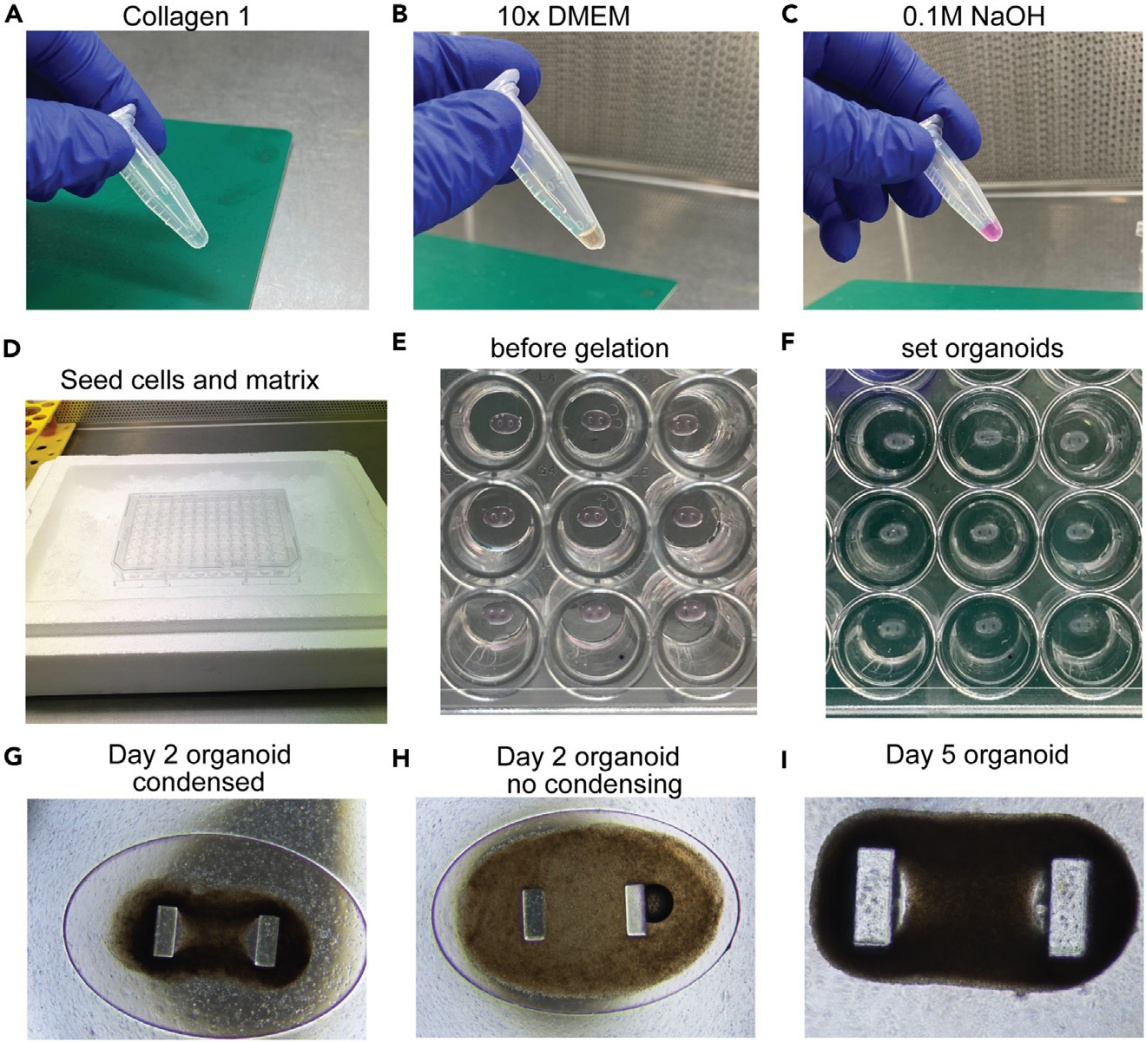
Preparation of human cardiac organoids (A) Acid solubilized Collagen 1 in Eppendorf tube. (B) Addition of 10x DMEM to Collagen, thoroughly mixed. (C) Addition of 0.1 M NaOH to matrix, thoroughly mixed. (D) PDMS organoid plate on ice in culture hood. (E) Example of organoid mixture in PDMS insert before gelation. Mixture is transparent and pink in color. (F) Example of organoid mixture in PDMS insert after gelation at 37°C. Mixture is opaque and white in color. (G) Example of organoid in culture after 2 days that has condensed, (H) had not condensed and (I) after 5 days in culture.
19. On ice, prepare organoid matrix mixture as outlined in ‘Organoid Matrix Mixture’ table using a P200 pipette tip:
  ***Note:*** Avoid introducing bubbles into the matrix mixture by slow pipetting and keeping the pipette tip under liquid surface.
  a. Prepare a 1.5 mL Eppendorf tube per 96 well plate mixture and place on ice.
  b. Aliquot collagen 1 slowly into 1.5 mL Eppendorf tube (volumes in ‘Organoid Matrix Mixture’ Table above). See Figure 2A for example.
  c. Add cold 10x DMEM and mix thoroughly with collagen using pipette tip (volumes in ‘Organoid Matrix Mixture’ Table above). See Figure 2B for example.
    ***Note:*** Color should change from clear to yellow to indicate solution is well mixed.
  d. Add 0.1 M NaOH and mix thoroughly (volumes in ‘Organoid Matrix Mixture’ Table above). See Figure 2C for example.
    ***Note:*** Color should change from yellow to pink to indicate solution is well mixed.
  e. Add Matrigel™ and mix thoroughly (volumes in ‘Organoid Matrix Mixture’ Table above).
    **CRITICAL:** Reagents should be added in the order listed above and thoroughly mixed with a P200 pipette tip at each step. All reagents and Eppendorf tubes should be kept on ice constantly.
20. Aspirate supernatant from cardiomyocyte and stromal cell pellet and re-suspend in cold serum free medium (volumes in ‘Organoid Matrix Mixture’ Table above).
21. Add cardiomyocyte and stromal cell suspension immediately to matrix mixture and mix thoroughly.

**CRITICAL:** Once cells and matrix are combined, proceed immediately to seeding mixture between the pillars in each well of the PDMS culture plate.

22. Place PDMS culture plate on ice. See Figure 2D for example.
23. Seed mixture into each well of the PDMS culture insert throughout the entire seeding reservoir (i.e., around and between the pillars covering the entire oval) (volumes in ‘Organoid Matrix Mixture’ Table above).

***Note:*** Tilt plate towards user and pipette mixture from bottom rows working up to ensure mixture in the plate stays cold prior to centrifugation.

24. Centrifuge plate 100 × *g* for 10 s with maximum acceleration and deceleration settings. See Figure 2E for example seeded plate.

***Note:*** Transport plate to the centrifuge on ice to reduce premature setting of collagen.

25. Remove plate from centrifuge and check even cell spread under the microscope (Zeiss Primovert, 10X objective).

***Note:*** Observe cell density and distribution around the pillars in several wells of the PDMS culture plate.

26. Return plate to incubator and store for ∼ 30 min at 37°C 5% CO2 until mixture turns opaque indicating that it has set. See Figure 2F for example.

**CRITICAL:** Matrix should set before adding medium to ensure mixture does not flush out of insert.

27. Add 150 μL per well of Serum Free medium (see table above).
28. Return plate to incubator (day 0 of cardiac organoid culture).

***Note:*** Cells will begin to condense the matrix around silicone pillars by 2 days after harvest. If no condensation has occurred after 7 days the organoids are unlikely to form. See Figures 2G–2I for examples of condensed and minimally condensed organoid after 2 days in culture and typical organoid at 5 days in culture.

29. On day 2 of organoid culture, aspirate medium and add 150 μL Maturation Medium per well (see table above). Medium is supplemented with 10 ng/mL PDGF-BB, 10 ng/mL FGF-2, and 33 μg/mL aprotinin.

***Note:*** Growth factors are added right before use.

30. On day 5 of organoid culture, aspirate medium and add 150 μL Maturation Medium per well (see table above). Medium is supplemented with 10 ng/mL PDGF-BB, 10 ng/mL FGF-2, and 33 μg/mL aprotinin.

***Note:*** Growth factors are added right before use.

31. On day 7 of organoid culture, aspirate medium and add 150 μL Weaning Medium per well (see table above).
32. On day 9 of organoid culture, aspirate medium and add 150 μL Weaning Medium per well (see table above).
33. On day 11 of organoid culture, aspirate medium and add 150 μL Weaning Medium per well (see table above).
34. On day 13 of organoid culture, proceed to immunofluorescence labeling or assessment of contractile force.

### Force analysis of human cardiac organoids

**Timing: 1 h**

This section describes the analysis of functional parameters of beating human cardiac organoids.

35. Switch on microscope with 4x objective and CO2 and temperature control.

***Note:*** Use automated stage controller e.g., Leica Thunder microscope and LAS X Life Science Microscope Software. Use a plate chamber for environmental control.

**CRITICAL:** Wait for temperature and CO2 to stabilize before commencing imaging.

**Figure 3:**
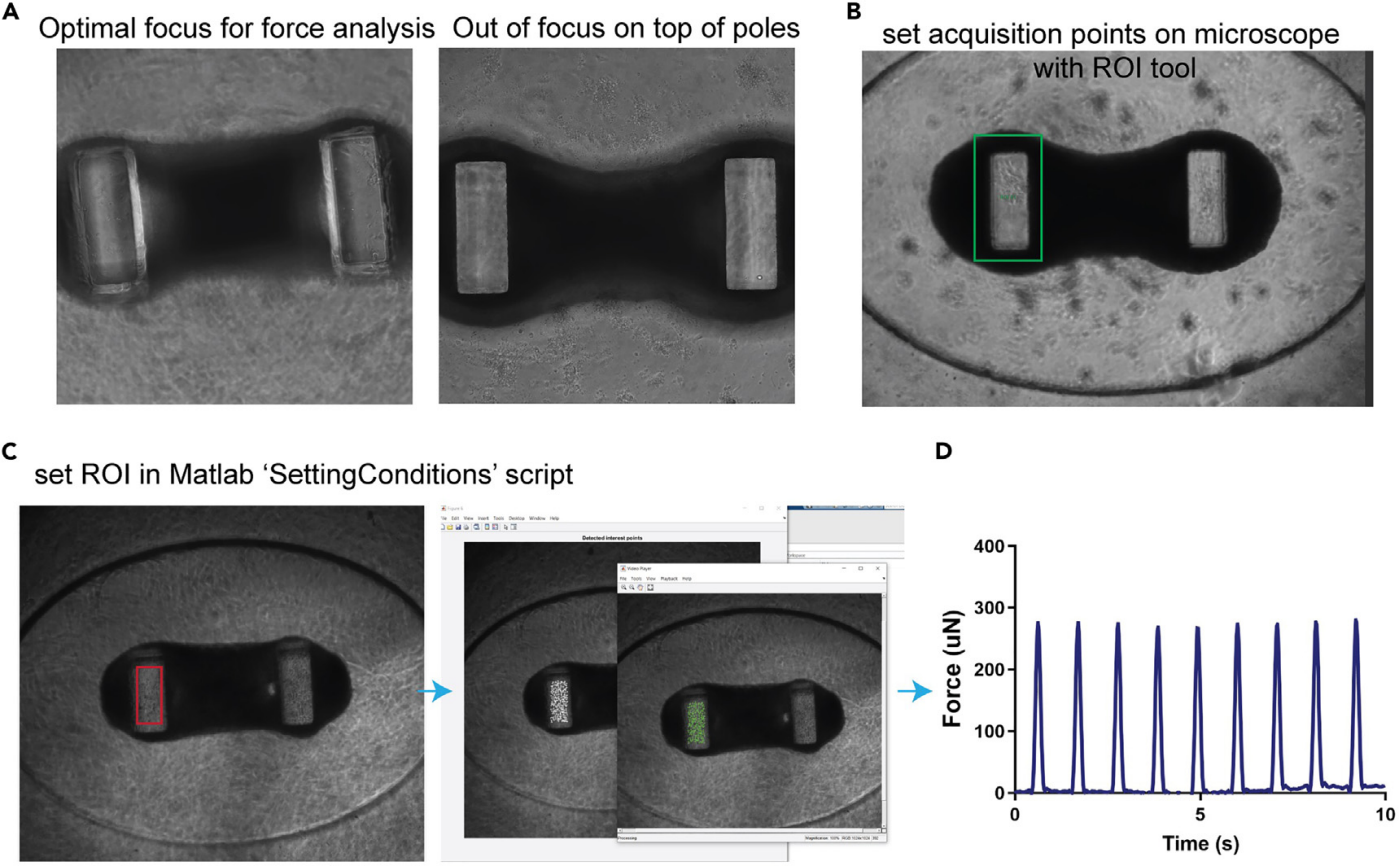
Workflow to convert image acquisition to force generation with Matlab (A) Bright-field image of organoid showing pole in focus (left) and out of focus (right). (B) Bright-field image of organoid with region of interest marker in acquisition software to align all following organoids. (C) Workflow for analyzing organoids with Matlab script. Define region of interest around the left pillar. (D) Example force trace graph generated by Matlab force analysis scripts.

***Note:*** In software, use square ROI in field of view to set consistent acquisition points within the frame (Figure 3B). Use low magnification (4x) to capture the whole organoid within the frame.

36. Set acquisition points for each organoid.
37. Using software control, focus Z axis on the top of the organoid pole (Figure 3A).

**CRITICAL:** Inaccurate focus on the top of the pole will influence downstream analysis of contractile force. Overexposure will result in loss of fine detail and color saturation which will negatively affect pixel detection and downstream contractile force analysis.

38. Set acquisition to record 500 frames over 10 s video (50 fps) with bright-field acquisition
39. Export multipage TIF files using naming system 001, 002 etc.

***Note:*** Files will be ∼250 MB per TIF.

40. Use MATLAB script ‘TIF2AVI’ to batch convert multipage TIF files into AVI video files (Matlab m-files are provided in Data S1). See Methods video S1: Example of contractile cardiac organoid.

***Note:*** Files will be on order of ∼10 MB per AVI.

**CRITICAL:** Ensure to download and install the Matlab plugins: Signal Processing Toolbox, Computer Vision Toolbox, Statistics and Machine Learning Toolbox, Image Processing Toolbox, Image Acquisition Toolbox, Bioinformatics Toolbox.

**CRITICAL:** Ensure files adopt naming system. For example ‘001’, ‘002’, ‘003’, etc.

41. Run ‘*SettingConditions’* Matlab script to read first video (Matlab m-files are provided in Data S1).
  a. Input ‘001.avi’ into line 3 within brackets.
42. Select ROI by drawing a box around left-hand pillar (Figure 3C).

***Note:*** Ensure pixels are detected as indicated by green crosses.

43. The script will generate coordinates for the ROI, copy these numbers into ‘*poletracking*’ Matlab script (Matlab m-files are provided in Data S1).

***Note:*** Can adjust *minpeakheight* (cut off for detecting beat) and *minpeakdistance* (cut off for the speed of beat) parameters depending on your particular experiment or platform. These are to help eliminate incorrectly assigned peaks.

44. Save changes.
45. Run *‘batchanalysis*’ Matlab script (Matlab m-files are provided in Data S1). This will generate a JPG file per video and an excel sheet with measurements of force contraction (Table 1).

**Table 1:** Example of raw force analysis analyzed with Matlab

Well number	Force (uN)	Rate (bpm)	Ta (s)	Tr (s)
1	267	36	0.15	0.14
2	315	36	0.16	0.13
3	258	36	0.15	0.13

***Note:*** The graphs for every video will be saved as part of running the ‘batchanalysis’ script. These should always be individually checked to ensure you do not have incorrectly assigned peaks which substantially skew the quantitative data.

Methods video S1. Example of contractile cardiac organoid, related to protocol step 40

### Immunolabeling of human cardiac organoids

**Timing: 2 days**

This section describes the process of fluorescence labeling of human cardiac organoids.

46. At the end of experiment, fix organoids in the plate:
  a. Aspirate culture medium.
  b. Add 50 μL of 1% PFA into each well.
  c. Fix at room temperature (15°C–25°C) for 60 min

***Note:*** PFA fixing can produce auto-fluorescence in cardiomyocytes.

47. Gently remove fixative from organoids and wash 2x with PBS.

**Pause point:** Fixed samples can be stored at 4°C for a week.

**Figure 4:**
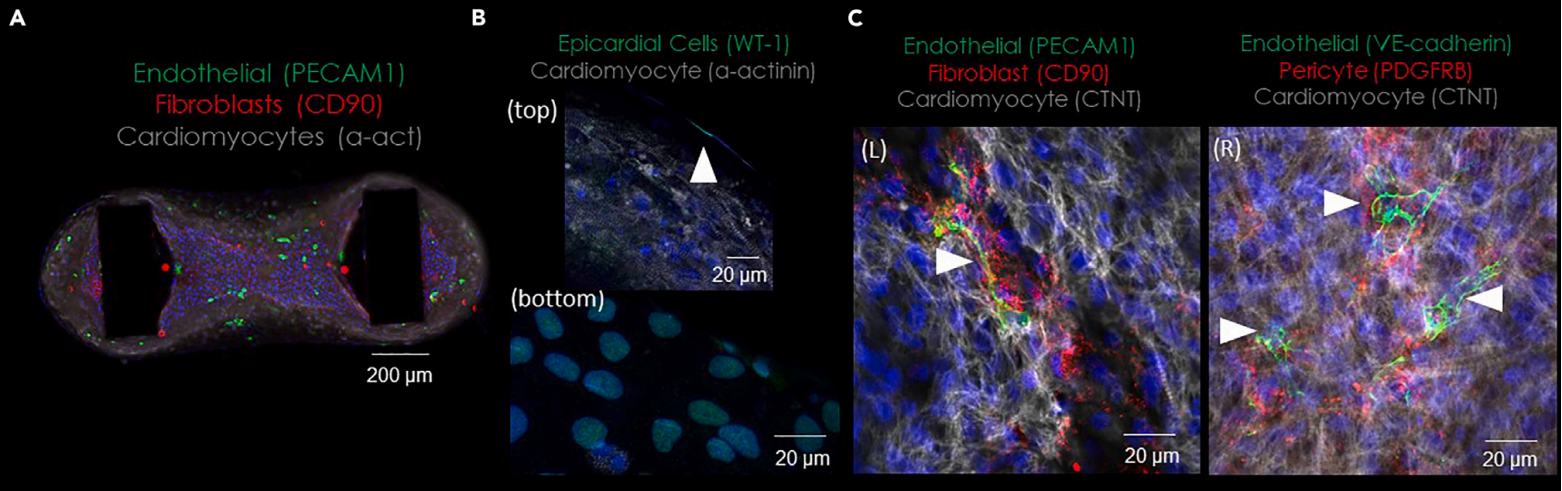
Representative immunohistochemical staining of organoids to show multicellularity (A) Whole-mount organoid stained with CD31 (endothelial cells), CD90 (stromal cells), α-actinin (cardiomyocytes) and Hoechst33342 (nuclei). (B) High magnification image of periphery of organoid stained with WT-1, α-actinin and Hoechst33342 at z-top (top) and z-middle (bottom). Arrows indicate formation of epicardial layer. (C) High magnification image of center of organoids stained with CD31, CD90, cTnT and Hoechst33342 (left) and VE cadherin, PDGFRβ, cTnT (right). Arrows indicate vessel formation.

***Note:*** Align replicate organoids with the same stain in a row on the slide for efficient imaging.

48. Gently remove PBS and add 100 μL blocking buffer (composition in Immunohistochemistry blocking buffer table) for 1–4 h at 4°C to permeabilize the sample.
49. Prepare primary antibody stains (see key resources table):
  a. On ice, add 100 μL blocking buffer to 1.5 mL Eppendorf tube.
  b. Add relevant primary antibodies.
    i. e.g., Mouse IgG1 CD31 (1:400); Mouse IgG2A CD90 (1:500); Rabbit cTnT (1:400) to blocking buffer.
50. Aspirate blocking buffer from organoids.
51. Add primary antibodies to organoids in the plate.
52. Incubate at 4°C on a rocker overnight (∼18 h) protected from evaporation using Parafilm and from light with aluminum foil.
53. Wash organoids:
  a. Aspirate primary antibodies.
  b. Add 100 μL of blocking buffer to organoids.
  c. Place on rocker at 4°C for 1 h.
  d. Aspirate blocking buffer.
  e. Add 100 μL of blocking buffer to organoids.
  f. Place on rocker at 4°C for 1 h.
54. Prepare secondary antibodies:
  a. On ice, add 100 μL of blocking buffer to 1.5 mL Eppendorf tube.
  b. Add relevant secondary antibodies.
    i. e.g., Goat anti-mouse IgG_1_ Alexa Fluor 488 (1:400); Goat anti-mouse IgG_2A_ 555 (1:400); Goat anti-rabbit (H&L) Alexa Fluor 633 (1:400 dilution); Hoechst33342 (1:1000).
  c. Protect from light.
55. Aspirate blocking buffer from organoids.
56. Add secondary antibodies to organoids in plate.
57. Incubate at 4°C on a rocker overnight (∼18 h) in the dark.
58. Wash organoids:
  a. Aspirate secondary antibodies.
  b. Add 100 μL of blocking buffer to organoids.
  c. Place on rocker at 4°C for 1 h.
  d. Aspirate blocking buffer.
  e. Add 100 μL of blocking buffer to organoids.
  f. Place on rocker at 4°C for 1 h.
59. Aspirate blocking buffer.
60. For organoids stained and to be imaged in the plate, proceed with tissue clearing protocol:
  a. Prepare tissue clearing solution: 60% (v/v) glycerol and 2.5M fructose in PBS.^4^
  b. Add 50 μL clearing solution per well.
  c. Image on fluorescence microscope with long working distance objectives e.g., Leica Thunder Microscope using 4x and 10x objectives.
61. For organoids imaged on glass slides, proceed with mounting:
  a. Prepare glass slide, coverslips and ProLongTM Glass Antifade Mountant.
  b. Using end of pipette tip, wedge the organoids from poles and place on glass slide.
  c. Add dropwise ProLongTM Glass Antifade Mountant on top of organoids.
  d. Carefully place coverslip over sample and ensure no bubbles form.
  e. Set overnight (∼18 h) at room temperature (15°C–25°C) before imaging.
62. Proceed to confocal microscope for fluorescence image acquisition. Example in Figure 4.

**Pause point:** Fixed samples can be stored protected from light at 4°C for 2 days.

## Expected outcomes

A differentiated culture is expected to generate multiple cardiac cell types and visually form a network of beating cardiomyocytes. The cellular composition of differentiated cardiac cells should be ∼70% cardiomyocytes and ∼30% stromal cells. A T25 is expected to generate 10–15 × 10^6^ cells at harvest. >95% Cardiac organoid formation efficiency is expected by 7 days post-harvest.

Cardiac organoids are expected to generate 200–500 μN of force. Cardiac organoids are expected to comprise of multiple cell types by day 7 that arise from the stromal fraction and self-sort into *in vivo* like morphologies, including cardiomyocytes (cTnT), stromal cells (CD90), endothelial cells (CD31), pericytes (NG2), and epicardial cells (WT1) (refer to Figure 4).

## Quantification and statistical analysis

Cardiac organoids were excluded from force analysis if the tissue was broken; a pole was broken or deformed; the tissue did not condense or the tissue had balled up or come off the pole.

## Limitations

Cardiac organoid generation relies on successful differentiation of human pluripotent stem cells. Some stem cell lines have reduced differentiation efficiency along some lineages and therefore the protocol may be unsuccessful. In our experience the consistency in growth, passaging, confluence prior to differentiation and high quality cultures with no partially differentiated cells overcomes limitations with particular cell lines. This is potentially the most variable and important step in making this protocol work successfully and generate the required multi-cellular populations.

## Troubleshooting

### Problem 1

The differentiation protocol to generate cardiac cell types does not produce 60–70% cardiomyocytes (protocol steps 1–5).

### Potential solution

Make sure to optimize initial seeding density for every hPSC line so that starting density on Day 0 is ∼70% confluent. hPSCs should also be adapted to feeder-free culture on Matrigel™ in mTeSR™ medium. Ensure growth factors are fresh.

### Problem 2

Cardiac organoids do not condense by day 7 (Figure 2G right hand side) (protocol steps 17–30).

### Potential solution

Ensure matrix is made up according to instructions and kept on ice during preparation. Look for the distinct color changes described above to indicate all reagents are working. Collagen1 will oxidize with continual opening of tubes, so aliquot from stock bottles into a working tube (∼5mL). 10x DMEM may precipitate out of solution, at which point it should be made fresh. Accurate cell counts are also required to generate the optimal cell to matrix ratio for the organoid to form. No condensation of organoids can also result with too much cell death following dissociation of the 2D differentiation, or because of the differentiation yielding very high cardiomyocyte proportions (see potential solution 1). Also ensure there is no residual BSA in the insert as this will also negatively impact and dilute the organoid matrix.

### Problem 3

Cardiac organoids lose structural integrity over culture time and break (protocol steps 29–34).

### Potential solution

This is usually due to insufficient matrix to support the cells in the organoid. Typically reagents in the ‘Organoid Matrix Mixture’ need replacing, the collagen I solution has formed a concentration gradient over long periods of time and the concentration is not accurate or the cardiac organoids do not contain the correct fibroblastic populations. Flow cytometry profiling is used to determine the ratio of fibroblasts to non-fibroblast cell populations.

### Problem 4

Cardiac organoids form but do not contract (protocol steps 35–38).

### Potential solution

This typically results from low cardiomyocyte fractions in the differentiation cultures, cardiomyocyte death during the dissociation or seeding phases and/or old/incorrectly formulated media.

## Resource availability

### Lead contact

Further information and requests for resources and reagents should be directed to and will be fulfilled by the lead contact, James Hudson, James.Hudson@qimrberghofer.edu.au.

### Materials availability

The specifications to generate the SU-8 wafer are attached (Data S2).

### Data and code availability

The codes generated during this study are attached. The published article^1^ and^2^ includes all datasets and code generated or analyzed during this study.

## Supplemental information

Data S1. Matlab files for contraction analysis, related to protocol steps 40–45

Data S2: SU-8 wafer CAD file accounting for PDMS shrinkage
